# Novel Selectively Targeted Multifunctional Nanostructured Lipid Carriers for Prostate Cancer Treatment

**DOI:** 10.3390/pharmaceutics14010088

**Published:** 2021-12-30

**Authors:** Lital Cohen, Yehuda G. Assaraf, Yoav D. Livney

**Affiliations:** 1 The Laboratory of Biopolymers for Food and Health, Department of Biotechnology and Food Engineering, Technion–Israel Institute of Technology, Haifa 3200003, Israel; litalco@campus.technion.ac.il; 2 The Fred Wyszkowski Cancer Research Laboratory, Department of Biology, Technion–Israel Institute of Technology, Haifa 3200003, Israel

**Keywords:** prostate cancer, nanoparticles, nanostructured lipid carriers, prostate-specific membrane antigen, targeted delivery, encapsulation, cabazitaxel

## Abstract

Prostate cancer (PC) is the most common cancer in men over 50 and the 4th most prevalent human malignancy. PC treatment may include surgery, androgen deprivation therapy, chemotherapy, and radiation therapy. However, the therapeutic efficacy of systemic chemotherapy is limited due to low drug solubility and insufficient tumor specificity, inflicting toxic side effects and frequently provoking the emergence of drug resistance. Towards the efficacious treatment of PC, we herein developed novel selectively PC-targeted nanoparticles (NPs) harboring a cytotoxic drug cargo. This delivery system is based upon PEGylated nanostructured lipid carriers (NLCs), decorated with a selective ligand, targeted to prostate-specific membrane antigen (PSMA). NPs loaded with cabazitaxel (CTX) displayed a remarkable loading capacity of 168 ± 3 mg drug/g SA-PEG, encapsulation efficiency of 67 ± 1%, and an average diameter of 159 ± 3 nm. The time-course of in vitro drug release from NPs revealed a substantial drug retention profile compared to the unencapsulated drug. These NPs were selectively internalized into target PC cells overexpressing PSMA, and displayed a dose-dependent growth inhibition compared to cells devoid of the PSMA receptor. Remarkably, these targeted NPs exhibited growth-inhibitory activity at pM CTX concentrations, being markedly more potent than the free drug. This selectively targeted nano-delivery platform bears the promise of enhanced efficacy and minimal untoward toxicity.

## 1. Introduction

**Figure 1:**
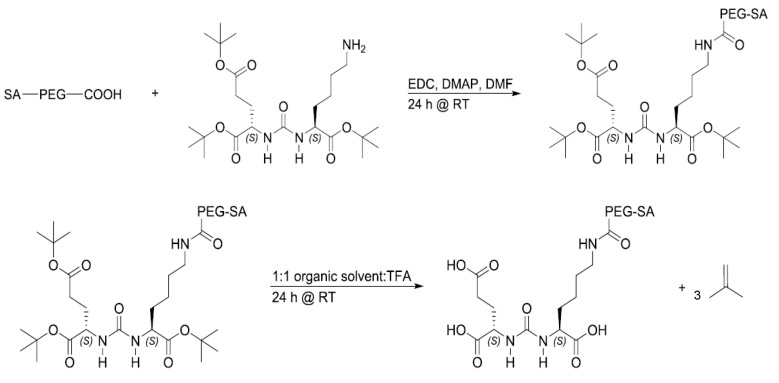
Synthesis of SA-PEG with PSMA TL Glu-Urea-Lys. SA-PEG-COOH was conjugated to Glu-Urea-Lys with protecting groups of tert-Butyl esters. The SA-PEG-TL was subjected to acidic conditions for the removal of the protecting groups, hence regaining the carboxylic groups of the PSMA TL.

Cancer is one of the leading causes of morbidity and mortality worldwide. In this respect, prostate cancer (PC) is the most common cancer in men over the age of 50 and the 4th most prevalent human malignancy [1]. The current modalities to treat early-stage PC include surgery, radiation therapy, and for some men, androgen deprivation therapy [2,3]. Unfortunately, however, PC cells frequently acquire resistance to therapy, resulting in local relapse, progression, and metastasis [2,3]. This latter phase of the disease is referred to as castration-resistant prostate cancer (CRPC) or metastatic CRPC (mCRPC), which can further become a lethal disease (see Figure 1 in reference [4]) [2,3,5]. The most common metastases reside in lymph nodes and bone [6]. The current treatment of CRPC includes systemic second-generation endocrine treatment, followed by combination chemotherapy, where docetaxel (DTX) and cabazitaxel (CTX) are the cytotoxic drugs approved for the treatment of this stage of the disease [6,7]. Like the parent drug paclitaxel (PTX), DTX and CTX are taxanes that promote microtubule assembly and inhibit microtubule depolymerization, thereby causing an antimitotic cytotoxic effect [8,9,10]. This treatment is limited by low drug bioavailability due to the poor solubility of these therapeutic agents in the blood and the side effects to healthy tissues, causing reduced quality of life [2,3,5,6,7]. The goal of cancer therapeutics is to achieve a curative targeted treatment which also reduces systemic toxicity [11].

One of the strategies to overcome these serious therapeutic hurdles and limitations is selective targeting using nanoparticles (NPs). The mode of drug accumulation and cytotoxicity relies on passive targeting, which depends on the enhanced permeability and retention (EPR) effect [11,12,13,14,15]. This effect is characterized by structural abnormalities in tumor vasculature and lack of lymphatic drainage. Thereby, it enables extravasation of NPs from the tumor’s leaky blood vessels into the tumor and results in marked accumulation in the internal tumor microenvironment (TME) [11,12,13,14,15]. The EPR effect provides favorable conditions for the use of long-circulating nanocarriers that can evade immune surveillance and interaction with the reticuloendothelial system (RES) [11,12,13,14,15]. The RES is a global system of macrophages, mainly in the liver and spleen, aimed at engulfing and eliminating foreign particles that are recognized by antibodies, a process known as opsonization [11,12,13,14,15]. Thereafter, actively targeted NPs interact with, and undergo, internalization into target cells via receptor-mediated endocytosis [11,12,13,14,15,16,17]. The mechanism underlying endocytosis is based upon the interaction between ligand-decorated NPs, and a tumor-specific antigen, or defined receptor, selectively overexpressed on the surface of PC cells.

**Figure 2:**
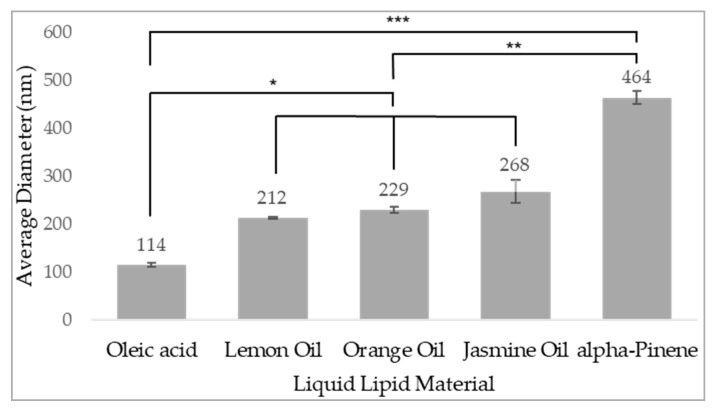
Mean diameter of monomodal distribution of NLCs with various liquid lipids. NLCs formulations of 60% SA-PEG and 40% of different liquid lipids were examined for size distribution. Values presented are means ± SE, n = 2 (two independent experiments each performed in triplicates). Significant differences between liquid lipids are marked as follows: * (*p* < 0.0080); ** (*p* < 0.0009); *** (*p* < 0.0005).

Various cell surface antigens were used for the targeting of PC cells via selective receptor-mediated endocytosis (see Figure 2 in reference [4]). One of the most highly selective and well-characterized biomarker antigens of PC is prostate-specific membrane antigen (PSMA), also known as glutamate carboxypeptidase II (GCP-II), N-acetyl-α-linked acidic dipeptidase I, or folate hydrolase [2,3]. PSMA is a surface receptor overexpressed by a factor of 100–1000 in 94% of PC cells, compared to normal tissues including healthy prostate epithelial tissues [2,3,18]. Moreover, its expression increases with cancer progression, aggressiveness, and metastasis [2,3,18]. Therefore, PSMA has been extensively used as a bona fide target antigen for both diagnostic imaging and targeted drug delivery in the treatment of PC [19,20,21,22,23,24]. Compounds based on a urea linkage between two amino acids were found to have comparable affinities for PSMA and a strong interaction with the active site of GCP-II via hydrogen bonds [25,26,27].

As NPs undergo internalization into target cells, intracellular drug release occurs, thereby achieving a specific therapeutic effect [11,12,13,14,15,16,17]. Furthermore, uptake via receptor-mediated endocytosis is usually followed by lysosomal degradation of the NPs, thus facilitating the release of the encapsulated drug cargo into the cytoplasm, hence achieving a potent therapeutic effect [11,12,13,14,15,16,17].

The selective internalization of targeted NPs is an effective strategy to overcome cancer multidrug resistance (MDR), which remains a primary impediment towards efficacious cancer therapy [15,28,29,30,31,32,33,34,35]. This selective targeting constitutes a major advantage to evade MDR efflux pumps including P-glycoprotein (Pgp/ABCB1), multidrug resistance-associated protein 1 (MRP1/ABCC1), and breast cancer resistance protein (BCRP/ABCG2); these ATP-driven transmembrane efflux pumps extrude a multitude of chemotherapeutic drugs which are structurally and mechanistically distinct, resulting in a broad spectrum resistance to multiple anticancer drugs known as MDR [15,36,37,38,39,40].

Several important physicochemical properties should be optimized to establish an effective nanocarrier system with regards to: (a) permeation out of the leaky blood vessels and accumulation in the TME through the EPR effect, and (b) specific interaction with target cells and intracellular release of the encapsulated drug. The size and zeta-potential of NPs are some of the most important factors to be considered. A size range of 50–200 nm was found to be suitable for efficient extravasation from the tumor blood vessels, avoiding filtration by the kidney and minimal capture by the liver [11,13]. NPs with negative or neutral zeta-potential, as an indicator for anionic or neutral surface charge, respectively, are less subjected to opsonization and are unlikely to undergo electrostatic interaction followed by non-specific uptake by the negatively charged cellular membranes [13].

As part of the innovative development of pharmaceutical drug carriers, nanostructured lipid carriers (NLCs) constitute an effective option for stable and controlled drug delivery. NLCs consist of a nanostructured lipid matrix, made of a mixture of solid and liquid lipids, surface-stabilized by a surfactant or a mixture of surfactants [41,42,43]. The incorporation of liquid lipids with solid lipids generates liquid domains within the crystalline structure of the solid lipid lattice, which in turn enables the stable entrapment of the lipophilic drug cargo for the enhancement of drug loading capacity and minimization of premature outward diffusion [41,42,43,44,45,46,47]. In the current study, we developed NLCs which are composed of biocompatible and biodegradable components, including an oleic acid liquid core, surface-stabilized by solidifying stearic acid (SA), conjugated to polyethylene glycol (SA-PEG). Coating the surface of NLCs with PEG, a process called PEGylation, provides stealth functionality (i.e., immune system “transparency”) that contributes to a markedly prolonged blood circulation time and is known to attenuate the clearance of the NPs from the circulation by hindering the otherwise dominant uptake by the RES [48]. This is critical for maximizing the passive uptake into the tumor and improving the pharmacokinetic and pharmacodynamic profiles of the nanocarriers. PEG is conjugated via an amide linkage to the targeting ligand (TL) Glutamate-Urea-Lysine (Glu-Urea-Lys), a small molecule urea-derivative which is an established ligand for specific binding to the PSMA receptor [25,26,27,49,50].

We undertook a thorough literature and patent review to confirm the novelty of the current nanodelivery system and delineate the differences of our nanomedicine system compared to other previously described NPs for prostate cancer treatment. A variety of nanocarrier drug delivery systems have been studied for the potential treatment of prostate cancer, including lipid-based NPs [51,52,53,54,55,56,57], inorganic NPs [58,59,60,61], and polymeric NPs [62,63,64,65,66,67,68]. Lipid NPs, including the NPs developed in our present study, have several advantages over other nanodelivery systems. The most compelling advantages of lipidic nanocarriers are their ease of scalability, low untoward toxicity, and more controllable release patterns [69]. Various lipidic NPs for prostate cancer treatment have been studied, and perhaps the most common lipidic drug delivery vehicles are liposomes. The major advantage of NLCs over liposomes is that the distinctive nanostructure of NLCs allows for a markedly enhanced loading capacity and sustained drug release profile. Hence, we chose NLCs for the current study.

We herein developed a targeted nanodelivery platform for the encapsulation of the therapeutic agent CTX. CTX is a novel second-generation anti-microtubule agent of the taxane family, with enhanced anti-tumor activity in MDR cancer cells, as, advantageously, it is a relatively poor efflux substrate of P-gp [70,71]. The current study aimed to construct a novel targeted drug nanodelivery system, characterize it, and evaluate the selective uptake and cytotoxicity to prostate cancer cells.

## 2. Materials and Methods

### 2.1. Materials

SA conjugated to polyethylene glycol (PEG) (2KDa) (SA-PEG) and SA-PEG with a carboxylic end group at the PEG terminus were custom-synthesized (Creative PEGWorks, Durham, NC). Glu-Urea-Lys with protecting groups of tert-Butyl esters was purchased from ABX advanced biochemical compounds (Radeberg, Germany). Cyanine7 with amine modification (Cy7-NH2) was purchased from Moshe Stauber Biotech Applications (Lod, Israel). All other chemicals were obtained from Sigma-Aldrich (Merck, Rehovot, Israel).

### 2.2. Cell Cultures

Human prostate cancer cell lines LNCaP (overexpressing the PSMA receptor) and PC-3 (devoid of PSMA expression), human non-small cell lung cancer (NSCLC) 1975 cells, human embryonic kidney HEK-293 cells, and normal human bronchial epithelial BEAS2B cells were cultured in an RPMI-1640 medium, supplemented with 10% fetal bovine serum (FBS), 2 mM glutamine, 100 μg/mL penicillin, and streptomycin (Biological Industries, Beit-HaEmek, Israel). The growth medium of LNCaP cells was supplemented with 5 μg/mL insulin (Sigma-Aldrich, Merck, Rehovot, Israel). Neonatal foreskin fibroblast FSE cells were cultured in a DMEM medium, supplemented with 10% FBS, 2 mM glutamine, 100 μg/mL penicillin, and streptomycin. Cells were incubated at 37 °C in a humidified atmosphere of 5% CO_2_. NSCLC 1975, HEK-293, BEAS2B, and FSE cells were obtained from ATCC [72]. LNCaP and PC-3 cells were generously provided by Prof. David (Dedi) Meiri (Technion, Haifa, Israel) and were also originally obtained from ATCC. The actual surface expression of the PSMA receptor by LNCaP and PC-3 cells was determined by immunohistochemistry, conducted by the department of pathology in Rambam Health Care Campus, Haifa, Israel.

### 2.3. Methods

#### 2.3.1. Preparation of NLCs

Self-assembled NLCs were prepared by a surfactant-free nanoprecipitation method [72,73,74] with several modifications for the stabilization of the nanocarriers system, as follows: For the unloaded NPs, SA-PEG (20 mg/mL) was dissolved in distilled water (DW) that was filtered through a 0.22 μm syringe filter prior to the addition of SA-PEG. The aqueous phase remained at 70 °C in order for the SA to be in its liquid state, as the melting point of SA was reported to be 69.6 °C [75]. A liquid lipid phase dissolved in ethanol (EtOH) was then added dropwise into the preheated aqueous phase under magnetic stirring of 600 rpm. The mixture was agitated at room temperature for 10 min to reach equilibrium. Different liquid lipids were used in the lipid phase including α-pinene, oleic acid (OA), orange oil, lemon oil, and jasmine oil. Samples were then transferred to a cold-water bath at 4 °C under stirring for another 10 min, for the solidification of the SA in the SA-PEG shell of the NPs, and in order to reach a final equilibrium.

#### 2.3.2. Synthesis of SA-PEG-TL and Preparation of Targeted NLCs

SA-PEG conjugated to PSMA targeting ligand (SA-PEG-TL) was synthesized by covalent coupling of SA-PEG-COOH with the amine group in the side chain of lysine in the PSMA TL, Glu-Urea-Lys (Figure 1) [76]. The carboxylic groups of Glu-Urea-Lys were masked by protecting groups of *tert*-Butyl esters. Briefly, SA-PEG-COOH (0.02 mmol), equal molar ratio of Glu-Urea-Lys TL (0.02 mmol), reagents 1-Ethyl-3-(3-dimethylaminopropyl)carbodiimide (EDC, 0.027 mmol), and 4-Dimethylaminopyridine (DMAP, 0.002 mmol) were dissolved in dimethylformamide (DMF, 1 mL), with continuous stirring at room temperature for 24 h. DMF was evaporated and ^1^H-NMR spectroscopy was conducted on a Bruker AVENCE II 400 MHz (^1^H at 400 MHz) instrument in DMSO-d_6_ as a solvent to validate the conjugation process [77]. Elimination of impurities was achieved by dialysis (molecular weight cutoff of 500–1000Da, BDL (Beith Dekel) Ltd., Raanana, Israel) against DW for 24 h. After conjugation and elimination of impurities, the SA-PEG-TL conjugation product was quantified using back titration. The excess of SA-PEG-COOH that did not react with the amine group on the PSMA TL was measured by titration with 0.01 M NaOH. The molarity of the SA-PEG-TL was calculated by the difference between the original molarity of SA-PEG-COOH and the molarity obtained by the acid-base titration. Thereafter, the PSMA TL protecting groups were removed under acidic conditions using trifluoroacetic acid (TFA) (Figure 1) and the isobutylene by-product was evaporated together with the solvents. ^1^H-NMR spectroscopy was conducted on a Bruker AVENCE 200 MHz (^1^H at 200 MHz) instrument in DMSO-d_6_ as a solvent to confirm the exposure of the carboxylic groups of the PSMA TL in the final SA-PEG-TL conjugation product [77]. For the preparation of targeted NLCs, SA-PEG-TL was added to the aqueous phase with SA-PEG at 70 °C. OA dissolved in EtOH was then added dropwise into the preheated aqueous phase under magnetic stirring of 600 rpm at room temperature for 10 min. Samples were then transferred to a cold-water bath at 4 °C under stirring for another 10 min.

#### 2.3.3. Preparation of Drug-Loaded NPs

Drug-loaded NPs were prepared as described in Section 2.3.1, with the addition of CTX dissolved in EtOH in the OA liquid lipid phase. The mixture was added dropwise to the water phase, containing SA-PEG and SA-PEG-TL. Samples were stirred at room temperature for 10 min and transferred to 4 °C for another 10 min. Samples were then centrifuged at 10,000× *g* at 4 °C for 20 min to sediment the unbound excess drug aggregates [72,73,74]. It was verified that under these centrifugation conditions the sediment does not contain any significant amount of the NPs, but only the crystalline drug excess.

#### 2.3.4. Particle Size Distribution and Zeta-Potential Analyses

The colloidal stability of the NLCs and the drug-loaded NPs was studied according to volume-weighted particle size distribution and zeta-potential using a dynamic light scattering (DLS)/zeta-potential analyzer, Zetasizer Ultra (Malvern Instruments Ltd., Worcestershire, UK). Samples were prepared by the nanoprecipitation method as described above and size distribution and zeta-potential measurements were carried out. Zeta-potential was calculated based on the Smoluchowski model [78]. Measurements from two independent experiments, each performed in triplicates, are presented as means ± standard error (SE).

#### 2.3.5. Analysis of Drug Loading Capacity and Encapsulation Efficiency

To quantify the amount of drug loaded into the NPs, NLCs were prepared at increasing drug to SA-PEG molar ratios [72,79]. The drug-loaded NPs were prepared and centrifuged as mentioned in Section 2.3.3 [72,73,74]. Quantification of the encapsulated drug in the supernatant was performed by lyophilizing the supernatant and dissolving it in EtOH to extract the CTX from the NLCs. Thereafter, the samples were filtered through a 0.45μm membrane. The concentration of drugs was determined using reversed-phase HPLC (RP-HPLC) and analyzed using a linear calibration curve [80,81]. RP-HPLC was conducted using a 4.6 × 250 mm C18 Kromasil RP-HPLC column and a UV detector at 234 nm for CTX detection. The mobile phase consisted of acetonitrile (ACN) and water (50:50, *v*/*v*). The injection volume was 20 μL, at a flow rate of 1ml/min. The column temperature was kept at 25 °C, and the HPLC run was set for 25 min [80,81] with a 6 min ACN rinse between runs.

Calculation of the loading capacity (LC, i.e., the mass ratio of drug to carrier) and encapsulation efficiency (EE) (i.e., the percent of encapsulated drug from the added drug) of the NPs was performed using Equations (1) and (2) [72,73,74]:

LC (mg drug/g SA-PEG) = W_ED_/W_SA-PEG_ (1)

EE (%) = (W_ED_/W_TD_) × 100% (2)

W_ED_ is the amount of encapsulated drug; W_SA-PEG_ is the total amount of SA-PEG in the sample; W_TD_ is the total amount of drug in the sample.

Results from two independent experiments, each performed in duplicates, are presented as means ± SE.

#### 2.3.6. Cryogenic-Transmission Electron Microscopy Imaging (Cryo-TEM)

Cryo-TEM (Philips CM120 microscope) analysis was used for the imaging of NLCs and NPs loaded with 0.6:1 CTX: SA-PEG molar ratio. Samples were prepared as described and images were taken at 4 °C. A Gatan Multi Scan 791 cooled CCD camera was used to acquire the images, using the Digital Micrograph 3.1 software package [72]. NPs size was determined using ImageJ software.

#### 2.3.7. Time-Course of In Vitro Drug Release

The time-course of in vitro release of drugs from the loaded NPs as compared to free drugs was studied by the dialysis method [72,73]. One ml of drug-loaded NPs or free drugs (both systems in DDW) were placed in dialysis tubes (molecular weight cutoff of 3.5 kDa, Sigma-Aldrich, Merck, Rehovot, Israel) and incubated in 30 mL of buffer comprising phosphate-buffered saline (PBS, 10 mM, pH 7.4) containing 0.1% wt Tween 80 for 72 h at 37 °C, with continuous gentle agitation (100 rpm). At defined time intervals (0, 2, 4, 6, 10, 23, 29, 36, 47, 58, 72 h), the dialysate was collected and replaced by the same volume of fresh release buffer. To calculate the cumulative release of drugs, the dialysates from each time point were freeze-dried, dissolved in EtOH for CTX extraction, filtered through 0.45 μm membrane, and quantified using RP-HPLC, as described in Section 2.3.5 above. The results are presented as the means of two independent experiments ± SE.

#### 2.3.8. Determination of NPs Concentration by Nanosight

NPs concentration was determined using Nanosight instrument (NS300, Malvern Instruments Ltd., Worcestershire, UK). Samples were prepared as described above, diluted with DW, and measured at 25 °C. Results are presented as the means of two independent experiments ± SE.

#### 2.3.9. Characterization of NPs Specificity by Confocal Laser Microscopy

Fluorescent NPs were formed as described above, with the addition of SA-PEG conjugated to the fluorophore Cy7 (SA-PEG-Cy7) in the aqueous phase during the NPs’ preparation. The conjugation between Cy7-NH_2_ and SA-PEG-COOH was performed using the EDC and N-hydroxysuccinimide (NHS) conjugation method as previously described [82]. SA-PEG-COOH (0.0125 mmol) was incubated with excess of EDC and NHS (at a 1:2 EDC: NHS molar ratio) for 15 min at room temperature with gentle shaking. The resulting NHS-activated SA-PEG was covalently linked to Cy7-NH_2_ (0.0125 mmol). The sample was allowed to react for 2 h with constant mixing at room temperature, and the final conjugate was dialyzed against DW (molecular weight cutoff of 3.5kDa, Sigma-Aldrich, Merck, Rehovot, Israel) for 24 h with five replacements of the dialysate to remove unreacted fluorophore.

Selective internalization of Cy7-labeled NPs was studied using confocal fluorescence microscopy (inverted confocal microscope, Zeiss LSM 710) [72,79]. Prior to the experiments, cells were seeded on μ-slides VI 0.4 (Ibidi, Martinsried, Germany) at 50% confluence and incubated overnight. Cells were then washed with PBS and incubated with 1 μg/mL Hoechst 33342 in growth medium for 10 min to achieve nuclear DNA staining. Thereafter, cells were incubated with different fluorescent NPs.

For selective internalization of targeted NPs as a function of the concentration of the PSMA TL, LNCaP target cells were incubated for 2 h at 37 °C, with non-targeted NPs and NPs decorated with increasing TL concentrations of 15 nM, 30 nM, and 80 nM (diluted 1:400 (*v*/*v*) in FBS-free medium). Thereafter, cells were washed twice with PBS to remove free fluorescent NPs. Two fluorescence channels were used during image capture: (1) blue for the viable DNA-dye Hoechst 33342 (Excitation/Emission: 350/461 nm); and (2) deep red for the Cy7-labeled NPs (Excitation/Emission: 720/750 nm).

To explore the internalization specificity of the NPs, LNCaP and PC-3 cells were incubated with non-targeted fluorescent NPs or 30 nM TL-decorated fluorescent NPs (diluted 1:400 (*v*/*v*) in FBS-free medium) for 2 h at 37 °C. In addition, NSCLC 1975, HEK293, BEAS2B, and FSE cells, were incubated with targeted fluorescent NPs (diluted 1:400 (*v*/*v*) in FBS-free medium) for 2 h at 37 °C. Then, cells were washed twice with PBS to remove free fluorescent NPs, and confocal microscope images were captured with the same fluorescence channels as mentioned above.

For characterization of the active internalization of NPs, LNCaP cells were incubated with a serum-free medium containing targeted NPs (diluted 1:400 (*v*/*v*)) for 1 h, at two different temperatures: 4 °C and 37 °C. Following incubation, cells were washed twice with PBS and the cellular fluorescence pattern was examined. Images were analyzed with IMARIS software.

#### 2.3.10. Growth Inhibition Assays

The selective growth inhibition of CTX-loaded NPs was studied in LNCaP target cells and PC-3 non-target cells, using an XTT (2,3-bis-(2-methoxy-4-nitro-5-sulfophenyl)-2H-tetrazolium-5-carboxanilide)-based colorimetric cell proliferation kit as previously described [72,79,83]. The preparation of CTX-loaded NPs was performed as described above and the NPs had undergone 24 h dialysis before cell exposure, to simulate conditions in the human body. The dialysis was conducted as described in Section 2.3.7, but with the use of 10% FBS to capture the released CTX [83]. Prior to the experiment, 96-well plates were coated with poly-L-Lysine before cell plating. LNCaP and PC-3 cells were seeded in 96-well plates at 5 × 10^4^ and 2.5 × 10^4^ cells/mL, respectively, and incubated overnight to allow for cell attachment. Following exposure of the cells to CTX-loaded NPs at increasing CTX concentrations of 0.001–100 nM for 24 h, cells were incubated for an additional 72 h with a fresh growth medium. Cellular growth inhibition was determined by adding the XTT reagent. Data were plotted using a nonlinear curve fitting of a sigmoidal model (Hill1) with OriginPro 9.0 to obtain a dose-response curve according to Equation (3) [72,79,83]:

P = P_∞_ + (P_0_ − P_∞_) × ([D]^n^/((IC_50_)^n^ + [D]^n^)) (3)

P stands for the percentage of live cells; P∞ represents the minimal percent of live cells at infinite drug concentration; P0 is the maximal percent of surviving cells in the absence of drug (100% = control); [D] stands for the drug concentration; IC50 stands for the drug concentration exerting 50% inhibition of cell growth; n indicates the abruptness of the dose-response curve.

Cells grown with free CTX at the same concentrations or medium with the addition of non-encapsulating NLCs served as the controls. Results shown are the means of two independent experiments, each performed in triplicates ± SE.

## 3. Results and Discussion

### 3.1. Self-Assembly of the Delivery System

NLCs are typically composed of a mixture of solid and liquid lipids at different ratios, designed to obtain stable core-shell (or multi-core-shell) structured spherical particles. We examined different formulations, and a mixture of 60% SA-PEG and 40% liquid lipid was selected for further characterization. Self-assembled NLCs were prepared as described in Section 2.3.1.

Various liquid lipids were studied for NP size distribution, as an assessment of the compatibility between the crystalline shell created by solidifying SA and the liquid lipid core (Figure 2).

To form a stable colloidal system of NPs suitable for effective accumulation in the TME and internalization into target cancer cells via endocytosis, we aimed at forming NPs with a mean diameter of 50–200 nm and a zeta-potential below −30mV (17, 19, 37). NPs with 40% oleic acid displayed a monomodal distribution with a mean size of less than 200 nm and were therefore selected as the formulation for further experiments.

**Figure 3:**
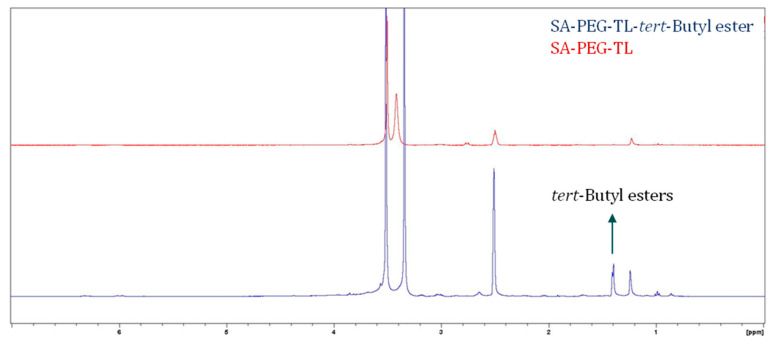
H-NMR spectroscopy. Tracings represent SA-PEG-TL with tert-Butyl esters protecting groups (blue) compared to the final conjugation product SA-PEG-TL (red). The H-NMR spectroscopy confirms that the tert-Butyl ester protecting groups were removed, thus revealing the carboxylic groups of the TL. Measurements were performed in duplicates.

To generate NPs that will selectively target PC cells, the PSMA TL, Glu-Urea-Lys, was conjugated via an amide linkage to SA-PEG. SA-PEG with a carboxylic end group at the PEG terminus and the amine group in the lysine side chain of the Glu-Urea-Lys TL with protecting groups of *tert*-Butyl esters, were conjugated for the synthesis of SA-PEG-TL (Figure 1). Following the conjugation and elimination of unwanted impurities, the protecting groups were removed to yield three carboxylic groups, constituting the binding motif to the PSMA receptor (Figure 1). NLCs with the PSMA TL (Targeted-NLCs) were slightly enlarged with an average diameter of 129 ± 3 nm, and a zeta-potential of −36.3 ± 0.3 mV. This negative surface charge of NPs was achieved thanks to the reduction of the carboxylic protecting groups of *tert*-Butyl esters, as shown in the H-NMR spectroscopy (Figure 3), thus exposing the negative charge of the TL at physiological pH. It is known that PEGylation (even without charge) provides good colloidal stability by steric repulsion of the NPs [84]. Moreover, the negative zeta potential provides additional colloidal stability by electrostatic repulsion, and it is also known that zeta potential that is more negative than (−30 mV), provides good colloidal stability [85] (even in the absence of PEGylation). Therefore, these results indicate that we have successfully formed colloidally stable NPs that could serve as a nanocarrier system that is decorated with a specific targeting ligand. This system was subjected to further analyses.

### 3.2. Physicochemical Characterization of NPs

**Figure 4:**
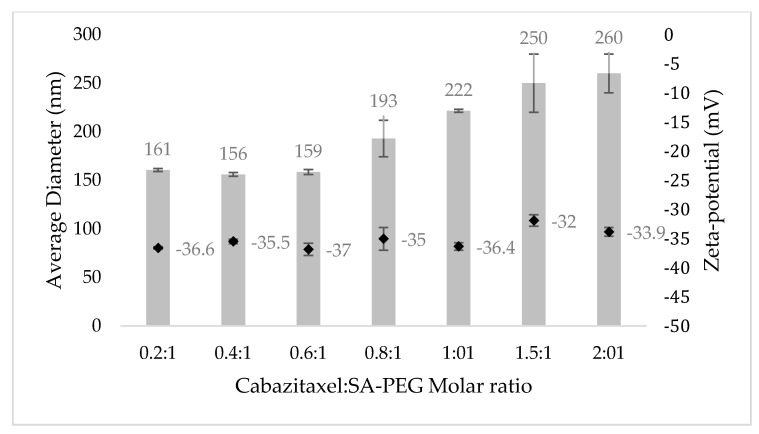
Mean diameter and zeta-potential of NPs at increasing CTX: SA-PEG molar ratios. Columns represent the mean diameter and black diamonds represent the zeta-potential. Values presented are means ± SE, n = 2 (two independent experiments, each performed in triplicates).

The size distribution and zeta-potential of targeted-NLCs were determined at increasing drug to SA-PEG molar ratios using DLS/zeta-potential analyzer [72,73,74,79]. CTX was dissolved in the liquid lipid phase together with the OA and loaded into the targeted NLCs by physical encapsulation. Following centrifugal sedimentation of the unbound excess drug, CTX-loaded NPs were studied for size distribution and displayed a monomodal distribution (Figure 4).

Expectedly, at increasing drug to SA-PEG molar ratios, there was an increase in the average diameter of NPs. This is due to the encapsulation of molecules that are characterized by a hydrophobic taxadiene core, as in the case of CTX. As the drug: SA-PEG ratio increased, the volume-to-surface ratio increased and thus the average diameter of the NPs increased. In contrast, the zeta-potential of the NPs remained negative and in the range of −35 ± 3 mV. As we aimed to obtain NPs with an average size of less than 200 nm, a CTX: SA-PEG molar ratio of 0.2–0.6:1 appeared to be suitable, and the drug loading capacity was hence studied.

### 3.3. Drug Loading Capacity (LC) and Encapsulation Efficiency (EE)

**Figure 5:**
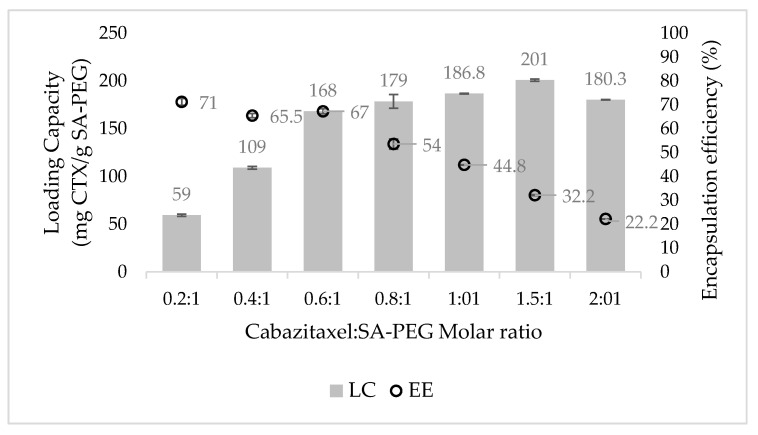
CTX loading capacity (LC) and encapsulation efficiency (EE) at increasing CTX: SA-PEG molar ratios. Columns represent the LC, whereas circles denote EE. Values shown are means ± SE, n = 2 (two independent experiments, each performed in triplicates).

The LC and EE of CTX at increasing drug to SA-PEG molar ratios were determined by centrifugal sedimentation of unbound excess drug and quantification of the encapsulated drug in the supernatant by RP-HPLC (Figure 5). Expectedly, at increasing drug to SA-PEG molar ratios, the EE decreased, whereas the LC increased. It is evident from Figure 5 that increased concentrations of CTX led to a significant increase in LC, up to 0.6:1 CTX to SA-PEG molar ratio. However, at higher drug concentrations, the increase in LC became more moderate. These results are consistent with the DLS data showing a major increase in the average diameter of NPs between 0.6:1 to 0.8:1 CTX: SA-PEG molar ratio (159 ± 3 nm and 193 ± 19 nm, respectively). Taken collectively, the formulation displaying the optimal relation between LC, EE, and particle size was 0.6:1 CTX: SA-PEG, with an average diameter of 159 ± 3 nm, LC of 168 ± 3 mg CTX/g SA-PEG, and EE of 67% ± 1%.

### 3.4. The Morphology of Drug-Loaded NPs

**Figure 6:**
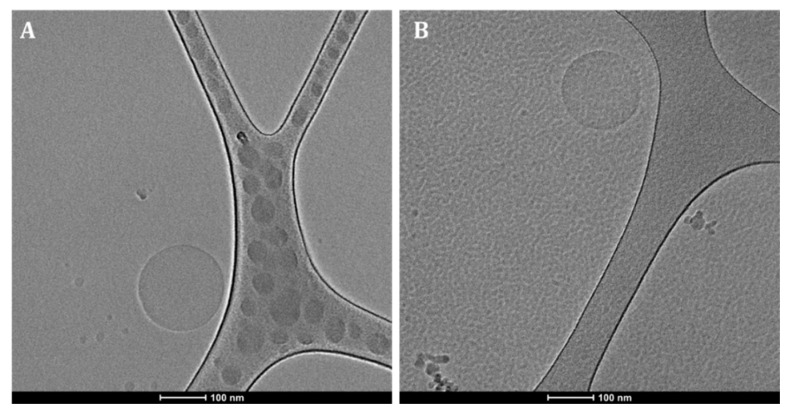
Analysis of the morphology of NPs by Cryo-TEM. Cryo-TEM images of (**A**) 0.6:1 CTX: SA-PEG NPs; and (**B**) NLCs not encapsulating a drug, in water. The scale bars denote 100 nm. Note that the CryoTEM images only show examples of the morphology of typical NPs. For size statistics, please refer to the DLS data.

The morphology of the NPs was examined by analyzing cryogenic transmission electron microscopy (Cryo-TEM) images (Figure 6). NPs loaded with CTX (Figure 6A) appear as spherical particles with an average diameter, which agrees with the DLS measurements. As it was previously found that spherical particles are able to undergo faster internalization than non-spherical NPs, this result contributes to the efficiency of the delivery system [86]. Additionally, non-encapsulated NLCs (Figure 6B) displayed a similar morphology, supporting the incorporation of drugs into the NLCs nanocapsules. 

### 3.5. In Vitro Profile of CTX Release from the NPs

The time-course of in vitro release of CTX from NPs was assessed by dialysis in a large volume of a PBS buffer pH 7.4 containing 0.1% wt Tween 80 for 72 h at 37 ℃, with continuous mixing. We used a buffer containing Tween 80 so that the hydrophobic drug released from the NPs was efficiently bound and solubilized by Tween 80%, mimicking human serum albumin binding of hydrophobic drugs. The above conditions were used to simulate the systemic circulation of NPs in the blood over 24–48 h, assuming this was a typical time for circulating NPs to accumulate in the tumor via the EPR effect [87]. At defined time intervals, the dialysate was replaced with fresh buffer, and the drug was quantified using RP-HPLC [72,73]. The complete replacement of fresh release buffer caused a stronger release driving force and constituted stringent release conditions [72,73].

**Figure 7:**
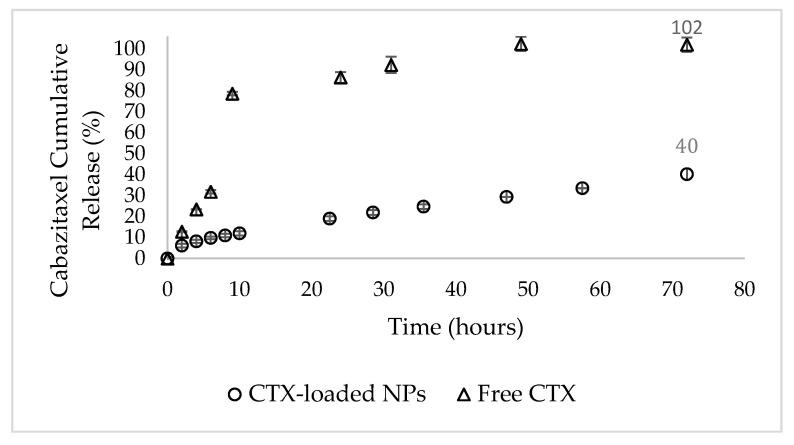
Time-course of CTX drug release. In vitro drug release profiles of CTX-loaded NPs (circles) compared to unencapsulated CTX (triangles). The values shown are means ± SE, n = 2 (two independent experiments).

The in vitro release profile of CTX from NPs showed substantial drug retention, as opposed to a burst-release of the unencapsulated drug during the first 10 h (Figure 7). These results were affected by the ratio between the liquid and solid lipids in the NPs formulation. As previously reported [41,42,43], the solid lipids of NLCs form a crystalline shell that acts as a barrier, and the liquid lipids form a cohesive hydrophobic inner core with strong interaction with the drugs loaded. Thus, a substantial reduction in drug release from the NPs was observed. However, the initial release of the drug from the NPs, possibly of some unencapsulated drug, or drug particles that were weakly adsorbed to the outer shell of the NPs, was slightly more rapid.

### 3.6. Selective Targeting and Internalization of NPs into LNCaP Target Cells

The selective internalization of targeted NPs labeled with the deep red fluorescent dye, Cy7, was studied using confocal laser microscopy. The cell lines that were examined were LNCaP, PC cells overexpressing PSMA that were used as the target cells; PC-3, rare PC cells lacking PSMA expression; NSCLC 1975 cells; human embryonic kidney HEK-293 cells; normal human bronchial epithelial BEAS2B cells; and neonatal foreskin fibroblast FSE cells.

**Figure 8:**
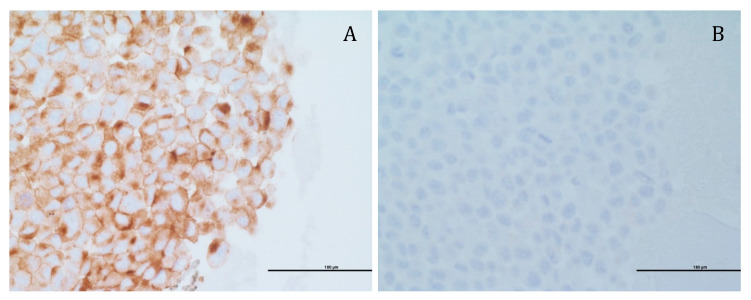
Immunohistochemistry analysis of PSMA expression. PSMA expression (shown as a brown diaminobenzidine immunohistochemistry staining) was examined in: (**A**) LNCaP; (**B**) PC-3 cell lines by the department of pathology in Rambam Health Care Campus. Scale bars denote 100 µm.

First, the actual surface expression of PSMA by the above PC cell lines was determined by immunohistochemistry, conducted by the department of pathology in Rambam Health Care Campus, Haifa, Israel. Expectedly, LNCaP cells displayed PSMA overexpression (Figure 8A), whereas PC-3 cells were devoid of PSMA (Figure 8B). These results are shown by the brown immunohistochemistry staining of the PSMA receptor that was largely confined to the plasma membrane of LNCaP cells.

**Figure 9:**
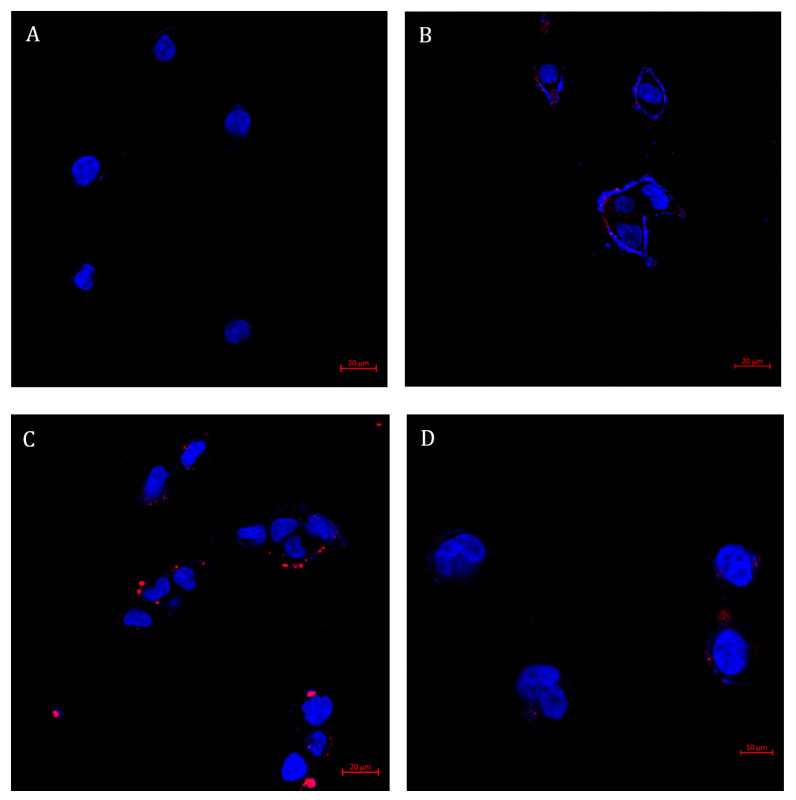
Selective targeting of NPs decorated with increasing concentrations of the TL, to PSMA-overexpressing LNCaP cells. Confocal laser microscopy images of LNCaP cells, following 2 h incubation with: (**A**) NPs lacking TL decoration; (**B**) NPs decorated with 15 nM of TL; (**C**) with 30 nM of TL; and (**D**) with 80 nM of TL (at 37 °C; samples were diluted 1:400 (*v*/*v*) in FBS-free medium). Nuclear DNA staining was achieved with Hoechst 33342 (1 μg/mL) and is marked in blue, whereas NPs labeled with Cy7 are marked in red. Scale bars denote 20 µm in (**A**–**C**), and 10 µm in (**D**).

The optimal amount of the TL conjugated to the NPs was examined by evaluation of the fluorescence signal in LNCaP target cells (Figure 9). The NPs were decorated with TL concentrations of 15 nM, 30 nM, and 80 nM, or no TL at all. We found that the internalization of NPs into LNCaP target cells is dependent on the concentration of TL decorating the NPs surface. Following 2 h incubation with NPs decorated with increasing TL concentrations at 37 °C, LNCaP cells displayed some cellular internalization upon 15 nM TL decoration and maximal internalization at 30 nM TL. The possible reasons for the result that optimal internalization of NPs occurred at 30 nM TL decoration, and only decreased at higher TL concentrations are: (a) A large number of TLs on the NPs surface may facilitate over-binding of a single NP to multiple PSMA receptors [88], thereby impairing proper internalization; (b) The large number of TLs sterically hindered these ligands from binding to the cell surface PSMA receptors [89]; (c) The negative surface charge of the NPs, caused by the large number of dissociated carboxylic groups on the PSMA TLs, resulted in an extensive repulsion between NPs and the negatively charged outer leaflet of the plasma membrane [13].

The observation of the complete lack of internalization upon exposure of target cells to non-targeted NPs confirmed that the targeting properties were specifically attributable to the PSMA TL, Glu-Urea-Lys. In conclusion, the targeted NPs selected for further studies were those decorated with 30 nM PSMA.

Determination of NPs concentration by Nanosight enabled to calculate the number of TL molecules on the NPs surface. NPs concentration was found to be 0.28894 ± 0.00007 nM, thus decoration of NPs with 30 nM TL resulted in ~100 TL molecules/NP.

**Figure 10:**
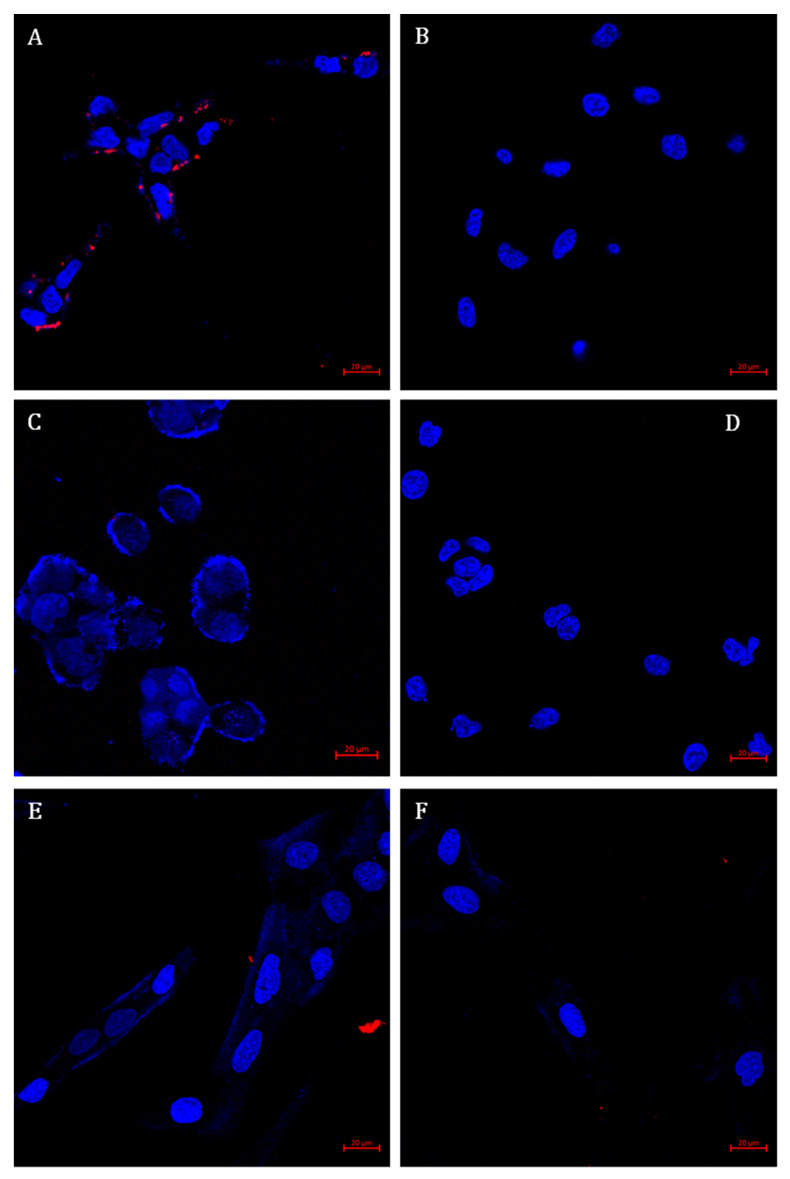
Specific internalization of targeted NPs. Confocal microscopy images of: (**A**) LNCaP; (**B**) PC-3; (**C**) NSCLC 1975; (**D**) HEK293; (**E**) BEAS2B; and (**F**) FSE cells, following 2 h incubation with targeted NPs labeled with Cy7 (at 37 °C; Samples were diluted 1:400 (*v*/*v*) in FBS-free medium). Nuclear DNA staining achieved with Hoechst 33342 (1 μg/mL) is marked in blue, whereas NPs labeled with Cy7 are marked in red. Scale bars denote 20 µm.

To verify the specific internalization of targeted NPs to LNCaP target cells via the PSMA receptor, different cell lines were examined including PC-3, NSCLC 1975, HEK-293, BEAS2B, and FSE cell lines (Figure 10).

Neither binding nor internalization were observed in non-target cells, demonstrating absolute specificity and enhanced internalization of NPs by PSMA-expressing PC cells. Moreover, the accumulation of targeted NPs within LNCaP cells appeared as perinuclear punctate staining. This suggests internalization via receptor-mediated endocytosis and intracellular accumulation in endolysosomes [90].

**Figure 11:**
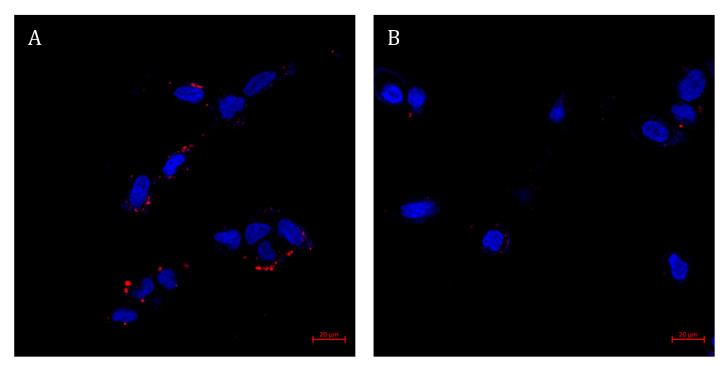
Characterization of active internalization of targeted NPs by LNCaP cells. Confocal laser microscopy images of LNCaP cells, following 1 h incubation with targeted NPs labeled with Cy7 (samples were diluted 1:400 (*v*/*v*) in FBS-free medium) at two different temperatures: (**A**) 37 °C; (**B**) 4 °C. Nuclear DNA staining achieved with Hoechst 33342 (1 μg/mL) is marked in blue, whereas NPs labeled with Cy7 are marked in red. Scale bars denote 20 µm.

For the characterization of active internalization by endocytosis, cells were incubated at two different temperatures: 4 °C and 37 °C. As endocytosis is an energy-dependent process, we assumed there will be higher accumulation at 37 °C, while at 4 °C only cell surface binding to the PSMA receptor may occur [90]. Confocal microscopy analysis (Figure 11) revealed that the uptake of targeted NPs was based on an endocytic process, with 17 red fluorescent dots per target LNCaP cell and an average intensity of 120 ± 60 (A.U.) at 37 °C compared to 4 dots per target cell and average intensity of 63 ± 30 (A.U.) at 4 °C. It should be noted that cellular processes other than endocytosis, such as diffusion, are also impeded at low temperatures and it is possible that TL binding to the PSMA receptor encouraged the diffusion of the NPs into the cell [91].

### 3.7. Selective Growth Inhibition of CTX-Loaded NPs In Vitro

**Figure 12:**
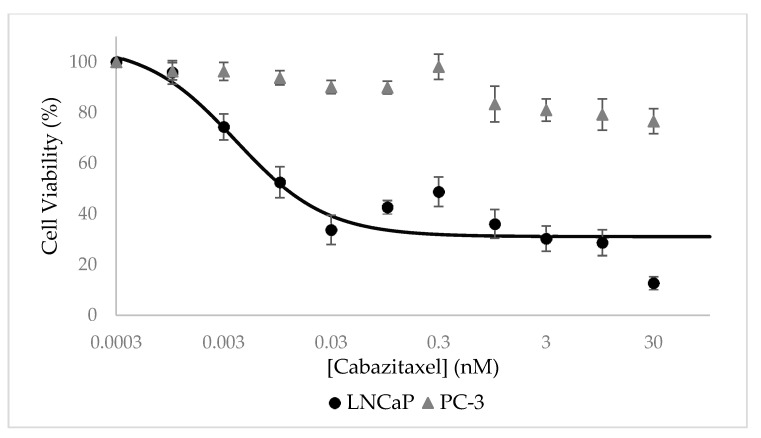
Selective growth inhibition of LNCaP target cells by CTX-loaded NPs. Cell growth inhibition as a function of the concentration of CTX encapsulated within NPs (0.6:1 CTX:SA-PEG molar ratio) in LNCaP target cells (circles) and non-target PC-3 cells (triangle). Values presented are means ± SE, n = 3 (three independent experiments, each performed in triplicates). The sigmoidal model curve of LNCaP cells was fitted using Equation (3).

Cytotoxicity assays were performed using an XTT-based colorimetric cell proliferation kit with PSMA-positive LNCaP cells and compared to PSMA-negative PC cells (PC-3). It is evident from Figure 12 that CTX-loaded NPs displayed a remarkable dose-dependent growth inhibitory effect on target LNCaP cells which overexpress PSMA, with an outstanding half-maximal inhibitory concentration (IC_50_) of 4.0 ± 2.0 pM. In contrast, these NPs did not exhibit any substantial growth inhibitory effect on PC-3 which lacked PSMA expression.

**Figure 13:**
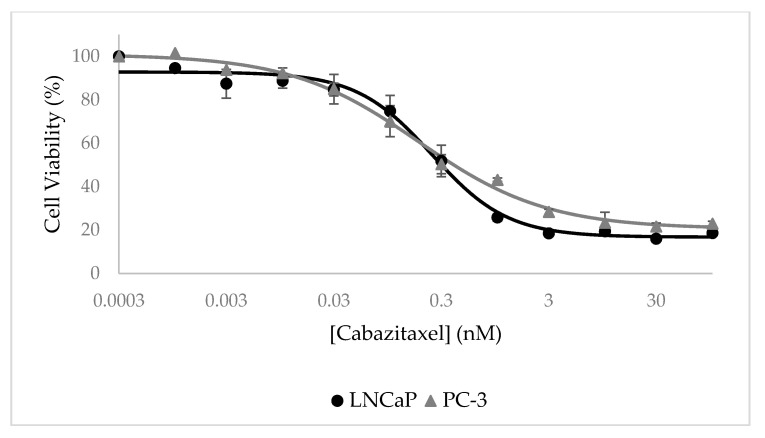
Inhibitory effect of free CTX. Cell viability as a function of free CTX concentration of LNCaP target cells (circles) and PC-3 non-target cells (triangle). Values presented are means ± SE, n = 3 (three independent experiments, each performed in triplicates). Sigmoidal model curves were fitted using Equation (3).

Cells exposed to free CTX in a growth medium for 72 h served as control and IC_50_ values obtained with LNCaP and PC-3 cells were similar: 0.25 ± 0.04 nM and 0.18 ± 0.04 nM, respectively (Figure 13).

The ratio of 62.5-fold between the IC_50_ values of free CTX and CTX-loaded NPs towards target LNCaP cells can be explained by the different exposure times and the targeting ability of the CTX-loaded NPs. The active targeting of CTX-loaded NPs enables the accumulation of NPs in target PC cells and thus a more potent anti-tumor activity. As a result, even at shorter exposure times (24 h, compared to 72 h of free CTX), the cytotoxic effect of the NPs was more significant against LNCaP target cells, as evident from the lower IC_50_ value. Moreover, it has been reported that the IC_50_ of free CTX exposure for 48 h towards LNCaP cells is 2.6–3.1 nM, which supports this conclusion [92,93]. Another important aspect that was achieved in the current study is the remarkable LC of 168 ± 3 mg CTX/g SA-PEG. In this respect, based on the concentration of NPs that was determined by Nanosight, we calculated that each NP carries a cargo of as much as ~700,000 CTX drug molecules. This remarkably large amount of potent cytotoxic drug molecules that are efficiently and specifically internalized by target PC cells, not only eliminate these tumor cells but may also kill neighbor PC cells upon apoptotic and lytic cell death. It is important to emphasize that drug-free NLCs were found to be non-toxic to cells.

## 4. Conclusions

Towards the efficacious treatment of PC, CRPC, and mCRPC, we herein developed novel selectively PC-targeted NPs harboring a cytotoxic drug cargo. These targeted NPs demonstrated efficient encapsulation of CTX, high specificity to, and potent eradication of, PSMA-positive target PC cells. Moreover, targeting NPs to the PSMA receptor resulted in uptake via receptor-mediated endocytosis, thus facilitating the intracellular release of the drug cargo, enabling the therapeutic activity. Another important aspect of selective internalization of these targeted NPs is the potential to overcome cancer MDR by evading MDR efflux pumps that extrude a broad spectrum of anticancer drugs from multidrug-resistant cancer cells. This selectively targeted nano-delivery platform should be able to enhance the efficacy of PC treatment, while reducing drug doses and minimizing untoward toxicity. Hence, these novel findings bear promise towards the improvement of patient survival rates and quality of life. Future in vivo studies are warranted that should assess the therapeutic efficacy of the targeted NPs against human PC in a xenograft murine model.

## Data Availability

The data presented in this study are available on request from the corresponding author.

## References

[1] Henley S.J., Ward E.M., Scott S., Ma J., Anderson R.N., Firth A.U., Thomas C.C., Islami F., Weir H.K., Lewis D.R. (2020). Annual report to the nation on the status of cancer, part I: National cancer statistics. Cancer.

[2] Jin W., Qin B., Chen Z., Liu H., Barve A., Cheng K. (2016). Discovery of PSMA-specific peptide ligands for targeted drug delivery. Int. J. Pharm..

[3] Barve A., Jin W., Cheng K. (2014). Prostate cancer relevant antigens and enzymes for targeted drug delivery. J. Control. Release.

[4] Cohen L., Livney Y.D., Assaraf Y.G. (2021). Targeted nanomedicine modalities for prostate cancer treatment. Drug Resist. Updates.

[5] Fujita K., Nonomura N. (2018). Role of Androgen Receptor in Prostate Cancer: A Review. World J. Men’s Health.

[6] Karantanos T., Evans C.P., Tombal B., Thompson T.C., Montironi R., Isaacs W.B. (2015). Understanding the mechanisms of androgen deprivation resistance in prostate cancer at the molecular level. Eur. Urol..

[7] Chen Y., Clegg N.J., Scher H.I. (2009). Anti-androgens and androgen-depleting therapies in prostate cancer: New agents for an established target. Lancet Oncol..

[8] Gelmon K. (1994). The taxoids: Paclitaxel and docetaxel. Lancet.

[9] Singla A.K., Garg A., Aggarwal D. (2002). Paclitaxel and its formulations. Int. J. Pharm..

[10] Haldar S., Chintapalli J., Croce C.M. (1996). Taxol induces bcl-2 phosphorylation and death of prostate cancer cells. Cancer Res..

[11] Danhier F., Feron O., Préat V. (2010). To exploit the tumor microenvironment: Passive and active tumor targeting of nanocarriers for anti-cancer drug delivery. J. Control. Release.

[12] Muhamad N., Plengsuriyakarn T., Na-Bangchang K. (2018). Application of active targeting nanoparticle delivery system for chemotherapeutic drugs and traditional/herbal medicines in cancer therapy: A systematic review. Int. J. Nanomedicine.

[13] Ernsting M.J., Murakami M., Roy A., Li S.-D.D. (2013). Factors controlling the pharmacokinetics, biodistribution and intratumoral penetration of nanoparticles. J. Control. Release.

[14] Torchilin V.P. (2010). Passive and active drug targeting: Drug delivery to tumors as an example. Handb. Exp. Pharmacol..

[15] Bar-Zeev M., Livney Y.D., Assaraf Y.G. (2017). Targeted nanomedicine for cancer therapeutics: Towards precision medicine overcoming drug resistance. Drug Resist. Updates.

[16] Maeda H., Wu J., Sawa T., Matsumura Y., Hori K. (2000). Tumor vascular permeability and the EPR effect in macromolecular therapeutics: A review. J. Control. Release.

[17] Belfiore L., Saunders D.N., Ranson M., Thurecht K.J., Storm G., Vine K.L. (2018). Towards clinical translation of ligand-functionalized liposomes in targeted cancer therapy: Challenges and opportunities. J. Control. Release.

[18] Minner S., Wittmer C., Graefen M., Salomon G., Steuber T., Haese A., Huland H., Bokemeyer C., Yekebas E., Dierlamm J. (2011). High level PSMA expression is associated with early psa recurrence in surgically treated prostate cancer. Prostate.

[19] Mayor N., Sathianathen N.J., Buteau J., Koschel S., Juanilla M.A., Kapoor J., Azad A., Hofman M.S., Murphy D.G. (2020). Prostate-specific membrane antigen theranostics in advanced prostate cancer: An evolving option. BJU Int..

[20] Jones W., Griffiths K., Barata P.C., Paller C.J. (2020). PSMA theranostics: Review of the current status of PSMA-targeted imaging and radioligand therapy. Cancers..

[21] Niaz M.O., Sun M., Ramirez-Fort M., Niaz M.J. (2020). Prostate-specific Membrane Antigen Based Antibody-Drug Conjugates for Metastatic Castration-resistance Prostate Cancer. Cureus.

[22] Niaz M.O., Sun M., Ramirez-Fort M., Niaz M.J. (2020). Review of Lutetium-177-labeled Anti-prostate-specific Membrane Antigen Monoclonal Antibody J591 for the Treatment of Metastatic Castration-resistant Prostate Cancer. Cureus.

[23] Iravani A., Violet J., Azad A., Hofman M.S. (2020). Lutetium-177 prostate-specific membrane antigen (PSMA) theranostics: Practical nuances and intricacies. Prostate Cancer Prostatic Dis..

[24] Chandran S.S., Ray S., Pomper M.G., Denmeade S.R., Mease R.C. (2016). Prostate Specific Membrane Antigen (PSMA) Targeted Nanoparticles for Therapy of Prostate Cancer. U.S. Patent.

[25] Kozikowski A.P., Zhang J., Nan F., Petukhov P.A., Grajkowska E., Wroblewski J.T., Yamamoto T., Bzdega T., Wroblewska B., Neale J.H. (2004). Synthesis of Urea-Based Inhibitors as Active Site Probes of Glutamate Carboxypeptidase II: Efficacy as Analgesic Agents. J. Med. Chem..

[26] Harada N., Kimura H., Ono M., Saji H. (2013). Preparation of asymmetric urea derivatives that target prostate-specific membrane antigen for SPECT imaging. J. Med. Chem..

[27] Chandran S.S., Banerjee S.R., Mease R.C., Pomper M.G., Denmeade S.R. (2008). Characterization of a targeted nanoparticle functionalized with a urea-based inhibitor of prostate-specific membrane antigen (PSMA). Cancer Biol. Ther..

[28] Lepeltier E., Rijo P., Rizzolio F., Popovtzer R., Petrikaite V., Assaraf Y.G., Passirani C. (2020). Nanomedicine to target multidrug resistant tumors. Drug Resist. Updates.

[29] Long L., Assaraf Y.G., Lei Z.N., Peng H., Yang L., Chen Z.S., Ren S. (2020). Genetic biomarkers of drug resistance: A compass of prognosis and targeted therapy in acute myeloid leukemia. Drug Resist. Updates.

[30] Nassir A.M., Ibrahim I.A.A., Md S., Waris M., Tanuja, Ain M.R., Ahmad I., Shahzad N. (2019). Surface functionalized folate targeted oleuropein nano-liposomes for prostate tumor targeting: In vitro and in vivo activity. Life Sci..

[31] Kopecka J., Trouillas P., Gašparović A.Č., Gazzano E., Assaraf Y.G., Riganti C. (2020). Phospholipids and cholesterol: Inducers of cancer multidrug resistance and therapeutic targets. Drug Resist. Updates.

[32] Assaraf Y.G., Brozovic A., Gonçalves A.C., Jurkovicova D., Linē A., Machuqueiro M., Saponara S., Sarmento-Ribeiro A.B., Xavier C.P.R., Vasconcelos M.H. (2019). The multi-factorial nature of clinical multidrug resistance in cancer. Drug Resist. Updates.

[33] Leonetti A., Wever B., Mazzaschi G., Assaraf Y.G., Rolfo C., Quaini F., Tiseo M., Giovannetti E. (2019). Molecular basis and rationale for combining immune checkpoint inhibitors with chemotherapy in non-small cell lung cancer. Drug Resist. Updates.

[34] Cui Q., Wang J.Q., Assaraf Y.G., Ren L., Gupta P., Wei L., Ashby C.R., Yang D.H., Chen Z.S. (2018). Modulating ROS to overcome multidrug resistance in cancer. Drug Resist. Updates.

[35] Jiang W., Xia J., Xie S., Zou R., Pan S., Wang Z.W., Assaraf Y.G., Zhu X. (2020). Long non-coding RNAs as a determinant of cancer drug resistance: Towards the overcoming of chemoresistance via modulation of lncRNAs. Drug Resist. Updates.

[36] Li W., Zhang H., Assaraf Y.G., Zhao K., Xu X., Xie J., Yang D.H., Chen Z.S. (2016). Overcoming ABC transporter-mediated multidrug resistance: Molecular mechanisms and novel therapeutic drug strategies. Drug Resist. Updates.

[37] Livney Y.D., Assaraf Y.G. (2013). Rationally designed nanovehicles to overcome cancer chemoresistance. Adv. Drug Deliv. Rev..

[38] Gottesman M.M., Lavi O., Hall M.D., Gillet J.P. (2016). Toward a Better Understanding of the Complexity of Cancer Drug Resistance. Annu. Rev. Pharmacol. Toxicol..

[39] Shapira A., Livney Y.D., Broxterman H.J., Assaraf Y.G. (2011). Nanomedicine for targeted cancer therapy: Towards the overcoming of drug resistance. Drug Resist. Updates.

[40] Zhang H., Xu H., Ashby C.R., Assaraf Y.G., Chen Z.S., Liu H.M. (2020). Chemical molecular-based approach to overcome multidrug resistance in cancer by targeting P-glycoprotein (P-gp). Med. Res. Rev..

[41] Beloqui A., Solinís M.Á., Rodríguez-Gascón A., Almeida A.J., Préat V. (2016). Nanostructured lipid carriers: Promising drug delivery systems for future clinics. Nanomed. Nanotechnol. Biol. Med..

[42] Selvamuthukumar S., Velmurugan R. (2012). Nanostructured Lipid Carriers: A potential drug carrier for cancer chemotherapy. Lipids Health Dis..

[43] Haider M., Abdin S.M., Kamal L., Orive G. (2020). Nanostructured lipid carriers for delivery of chemotherapeutics: A review. Pharmaceutics.

[44] Emami J., Rezazadeh M., Varshosaz J., Tabbakhian M., Aslani A. (2012). Formulation of LDL Targeted Nanostructured Lipid Carriers Loaded with Paclitaxel: A Detailed Study of Preparation, Freeze Drying Condition, and In Vitro Cytotoxicity. J. Nanomater..

[45] Chen Y., Pan L., Jiang M., Li D., Jin L. (2016). Nanostructured lipid carriers enhance the bioavailability and brain cancer inhibitory efficacy of curcumin both in vitro and in vivo. Drug Deliv..

[46] Jiang H., Geng D., Liu H., Li Z., Cao J. (2016). Co-delivery of etoposide and curcumin by lipid nanoparticulate drug delivery system for the treatment of gastric tumors. Drug Deliv..

[47] Bin Z., Yueying Z., Yu D. (2017). Lung cancer gene therapy: Transferrin and hyaluronic acid dual ligand-decorated novel lipid carriers for targeted gene delivery. Oncol. Rep..

[48] Lin W.J., Juang L.W., Lin C.C. (2003). Stability and release performance of a series of pegylated copolymeric micelles. Pharm. Res..

[49] Zhang Z., Zhu Z., Yang D., Fan W., Wang J., Li X., Chen X., Wang Q., Song X. (2016). Preparation and affinity identification of glutamic acid-urea small molecule analogs in prostate cancer. Oncol. Lett..

[50] Maresca K.P., Hillier S.M., Femia F.J., Keith D., Barone C., Joyal J.L., Zimmerman C.N., Kozikowski A.P., Barrett J.A., Eckelman W.C. (2009). A Series of Halogenated Heterodimeric Inhibitors of Prostate Specific Membrane Antigen (PSMA) as Radiolabeled Probes for Targeting Prostate Cancer. J. Med. Chem..

[51] Pereira S.G.T., Hudoklin S.S., Kreft M.E., Kostevsek N., Stuart M.C.A., Al-Jamal W.T. (2019). Intracellular Activation of a Prostate Specific Antigen-Cleavable Doxorubicin Prodrug: A Key Feature toward Prodrug-Nanomedicine Design. Mol. Pharm..

[52] Wang L., Qu M., Huang S., Fu Y., Yang L., He S., Li L., Zhang Z., Lin Q., Zhang L. (2018). A novel α-enolase-targeted drug delivery system for high efficacy prostate cancer therapy. Nanoscale.

[53] Ikemoto K., Shimizu K., Ohashi K., Takeuchi Y., Shimizu M., Oku N. (2016). Bauhinia purprea agglutinin-modified liposomes for human prostate cancer treatment. Cancer Sci..

[54] Cao Y., Zhou Y., Zhuang Q., Cui L., Xu X., Xu R., He X. (2015). Anti-tumor effect of RGD modified PTX loaded liposome on prostatic cancer. Int. J. Clin. Exp. Med..

[55] Zhang L., Shan X., Meng X., Gu T., Lu Q., Zhang J., Chen J., Jiang Q., Ning X. (2019). The first integrins β3-mediated cellular and nuclear targeting therapeutics for prostate cancer. Biomaterials.

[56] Patil Y., Shmeeda H., Amitay Y., Ohana P., Kumar S., Gabizon A. (2018). Targeting of folate-conjugated liposomes with co-entrapped drugs to prostate cancer cells via prostate-specific membrane antigen (PSMA). Nanomedicine.

[57] EP3799888A1—Liposomes Comprising Anti-Lox Antibody. https://www.patentguru.com/EP3799888A1.

[58] Saroj S., Rajput S.J. (2018). Etoposide encased folic acid adorned mesoporous silica nanoparticles as potent nanovehicles for enhanced prostate cancer therapy: Synthesis, characterization, cellular uptake and biodistribution. Artif. Cells Nanomed. Biotechnol..

[59] Tambe P., Kumar P., Paknikar K.M., Gajbhiye V. (2018). Decapeptide functionalized targeted mesoporous silica nanoparticles with doxorubicin exhibit enhanced apoptotic effect in breast and prostate cancer cells. Int. J. Nanomed..

[60] Rivero-Buceta E., Vidaurre-Agut C., Vera-Donoso C.D., Benlloch J.M., Moreno-Manzano V., Botella P. (2019). PSMA-Targeted Mesoporous Silica Nanoparticles for Selective Intracellular Delivery of Docetaxel in Prostate Cancer Cells. ACS Omega.

[61] Kumar A., Huo S., Zhang X., Liu J., Tan A., Li S., Jin S., Xue X., Zhao Y., Ji T. (2014). Neuropilin-1-targeted gold nanoparticles enhance therapeutic efficacy of platinum(IV) drug for prostate cancer treatment. ACS Nano.

[62] Hrkach J., Von Hoff D., Ali M.M., Andrianova E., Auer J., Campbell T., De Witt D., Figa M., Figueiredo M., Horhota A. (2012). Preclinical development and clinical translation of a PSMA-targeted docetaxel nanoparticle with a differentiated pharmacological profile. Sci. Transl. Med..

[63] Von Hoff D.D., Mita M.M., Ramanathan R.K., Weiss G.J., Mita A.C., Lorusso P.M., Burris H.A., Hart L.L., Low S.C., Parsons D.M. (2016). Phase I study of PSMA-targeted docetaxel-containing nanoparticle BIND-014 in patients with advanced solid tumors. Clin. Cancer Res..

[64] Autio K.A., Dreicer R., Anderson J., Garcia J.A., Alva A., Hart L.L., Milowsky M.I., Posadas E.M., Ryan C.J., Graf R.P. (2018). Safety and Efficacy of BIND-014, a Docetaxel Nanoparticle Targeting Prostate-Specific Membrane Antigen for Patients with Metastatic Castration-Resistant Prostate Cancer: A Phase 2 Clinical Trial. JAMA Oncol..

[65] Autio K.A., Garcia J.A., Alva A.S., Hart L.L., Milowsky M.I., Posadas E.M., Ryan C.J., Summa J.M., Youssoufian H., Scher H.I. (2016). A phase 2 study of BIND-014 (PSMA-targeted docetaxel nanoparticle) administered to patients with chemotherapy-naïve metastatic castration-resistant prostate cancer (mCRPC). J. Clin. Oncol..

[66] Bharali D.J., Sudha T., Cui H., Mian B.M., Mousa S.A. (2017). Anti-CD24 nano-targeted delivery of docetaxel for the treatment of prostate cancer. Nanomed. Nanotechnol. Biol. Med..

[67] Chen Z., Tai Z., Gu F., Hu C., Zhu Q., Gao S. (2016). Aptamer-mediated delivery of docetaxel to prostate cancer through polymeric nanoparticles for enhancement of antitumor efficacy. Eur. J. Pharm. Biopharm..

[68] Karandish F., Haldar M.K., You S., Brooks A.E., Brooks B.D., Guo B., Choi Y., Mallik S. (2016). Prostate-Specific Membrane Antigen Targeted Polymersomes for Delivering Mocetinostat and Docetaxel to Prostate Cancer Cell Spheroids. ACS Omega.

[69] Ghasemiyeh P., Mohammadi-Samani S. (2018). Solid lipid nanoparticles and nanostructured lipid carriers as novel drug delivery systems: Applications, advantages and disadvantages. Res. Pharm. Sci..

[70] Yin X., Luo L., Li W., Yang J., Zhu C., Jiang M., Qin B., Yuan X., Yin H., Lu Y. (2019). A cabazitaxel liposome for increased solubility, enhanced antitumor effect and reduced systemic toxicity. Asian J. Pharm. Sci..

[71] Sun B., Straubinger R.M., Lovell J.F. (2018). Current taxane formulations and emerging cabazitaxel delivery systems. Nano Res..

[72] Engelberg S., Netzer E., Assaraf Y.G., Livney Y.D. (2019). Selective eradication of human non-small cell lung cancer cells using aptamer-decorated nanoparticles harboring a cytotoxic drug cargo. Cell Death Dis..

[73] Zhao X., Tang D., Yang T., Wang C. (2018). Facile preparation of biocompatible nanostructured lipid carrier with ultra-small size as a tumor-penetration delivery system. Colloids Surf. B Biointerfaces.

[74] Ding X., Xu X., Zhao Y., Zhang L., Yu Y., Huang F., Yin D., Huang H. (2017). Tumor targeted nanostructured lipid carrier co-delivering paclitaxel and indocyanine green for laser triggered synergetic therapy of cancer. RSC Adv..

[75] Severino P., Pinho S.C., Souto E.B., Santana M.H.A. (2011). Polymorphism, crystallinity and hydrophilic–lipophilic balance of stearic acid and stearic acid–capric/caprylic triglyceride matrices for production of stable nanoparticles. Colloids Surf. B Biointerfaces.

[76] Malhotra M., Tomaro-Duchesneau C., Prakash S. (2013). Synthesis of TAT peptide-tagged PEGylated chitosan nanoparticles for siRNA delivery targeting neurodegenerative diseases. Biomaterials.

[77] Fulmer G.R., Miller A.J.M., Sherden N.H., Gottlieb H.E., Nudelman A., Stoltz B.M., Bercaw J.E., Goldberg K.I. (2010). NMR chemical shifts of trace impurities: Common laboratory solvents, organics, and gases in deuterated solvents relevant to the organometallic chemist. Organometallics.

[78] Delgado A.V., González-Caballero F., Hunter R.J., Koopal L.K., Lyklema J. (2005). Measurement and Interpretation of Electrokinetic Phenomena (IUPAC Technical Report). Pure Appl. Chem..

[79] Edelman R., Assaraf Y.G., Levitzky I., Shahar T., Livney Y.D. (2017). Hyaluronic acid-serum albumin conjugate-based nanoparticles for targeted cancer therapy. Oncotarget.

[80] Xin H., Chen L., Gu J., Ren X., Wei Z., Luo J., Chen Y., Jiang X., Sha X., Fang X. (2010). Enhanced anti-glioblastoma efficacy by PTX-loaded PEGylated poly(ε-caprolactone) nanoparticles: In vitro and in vivo evaluation. Int. J. Pharm..

[81] Qu N., Lee R.J., Sun Y., Cai G., Wang J., Wang M., Lu J., Meng Q., Teng L., Wang D. (2016). Cabazitaxel-loaded human serum albumin nanoparticles as a therapeutic agent against prostate cancer. Int. J. Nanomedicine.

[82] Aravind A., Varghese S.H., Veeranarayanan S., Mathew A., Nagaoka Y., Iwai S., Fukuda T., Hasumura T., Yoshida Y., Maekawa T. (2012). Aptamer-labeled PLGA nanoparticles for targeting cancer cells. Cancer Nanotechnol..

[83] Engelberg S., Lin Y., Assaraf Y.G., Livney Y.D. (2021). Targeted nanoparticles harboring jasmine-oil-entrapped paclitaxel for elimination of lung cancer cells. Int. J. Mol. Sci..

[84] Qian W., Murakami M., Ichikawa Y., Che Y. (2011). Highly efficient and controllable PEGylation of gold nanoparticles prepared by femtosecond laser ablation in water. J. Phys. Chem. C.

[85] Standard A. (1985). ASTM D4187-82: Zeta Potential of Colloids in Water and Waste Water.

[86] Champion J.A., Mitragotri S. (2006). Role of target geometry in phagocytosis. Proc. Natl. Acad. Sci. USA.

[87] Yaari Z., Da Silva D., Zinger A., Goldman E., Kajal A., Tshuva R., Barak E., Dahan N., Hershkovitz D., Goldfeder M. (2016). Theranostic barcoded nanoparticles for personalized cancer medicine. Nat. Commun..

[88] Zhao H., Yung L.Y.L. (2008). Selectivity of folate conjugated polymer micelles against different tumor cells. Int. J. Pharm..

[89] Wang M., Thanou M. (2010). Targeting nanoparticles to cancer. Pharmacol. Res..

[90] Harush-Frenkel O., Debotton N., Benita S., Altschuler Y. (2007). Targeting of nanoparticles to the clathrin-mediated endocytic pathway. Biochem. Biophys. Res. Commun..

[91] Dreifuss T., Ben-Gal T.-S., Shamalov K., Weiss A., Jacob A., Sadan T., Motiei M., Popovtzer R. (2018). Uptake mechanism of metabolic-targeted gold nanoparticles. Nanomedicine.

[92] Sekino Y., Han X., Kawaguchi T., Babasaki T., Goto K., Inoue S., Hayashi T., Teishima J., Shiota M., Yasui W. (2019). TUBB3 Reverses Resistance to Docetaxel and Cabazitaxel in Prostate Cancer. Int. J. Mol. Sci..

[93] Al Nakouzi N., Le Moulec S., Albigès L., Wang C., Beuzeboc P., Gross-Goupil M., De La Motte Rouge T., Guillot A., Gajda D., Massard C. (2015). Cabazitaxel Remains Active in Patients Progressing After Docetaxel Followed by Novel Androgen Receptor Pathway Targeted Therapies. Eur. Urol..

